# A119 CLINICAL MANAGEMENT OF PATIENTS WITH COLORECTAL INTRAMUCOSAL CARCINOMA COMPARED TO HIGH-GRADE DYSPLASIA AND T1 COLORECTAL CANCER

**DOI:** 10.1093/jcag/gwae059.119

**Published:** 2025-02-10

**Authors:** E Medawar, R Djinbachian, D Rex, M Vieth, H Pohl, I Popescu Crainic, M Taghiakbari, P Marques, D Kaufman, F Huang, D von Renteln

**Affiliations:** University of Ottawa, Ottawa, ON, Canada; Universite de Montreal, Montreal, QC, Canada; Indiana University School of Medicine, Indianapolis, IN; Friedrich-Alexander-Universitat Erlangen-Nurnberg, Bayreuth, Bayern, Germany; White River Junction VA Medical Center, White River Junction, VT; Universite de Montreal, Montreal, QC, Canada; Universite de Montreal, Montreal, QC, Canada; McMaster University, Hamilton, ON, Canada; Universite de Montreal, Montreal, QC, Canada; Universite de Montreal, Montreal, QC, Canada; Universite de Montreal, Montreal, QC, Canada

## Abstract

**Background:**

In the colorectum, intramucosal carcinoma (IMC), like high-grade dysplasia (HGD), should be resected endoscopically.

**Aims:**

We were interested to understand how real-world treatment of IMC cases compares to the management of HGD and T1 colorectal cancer (CRC).

**Methods:**

A multicenter retrospective cohort study was conducted. Through pathology databases, all patients diagnosed between 2010 and 2019 with HGD, IMC or T1 CRC were identified. Pathology and endoscopy reports were verified for HGD, IMC or T1 CRC polyps. The primary outcome was the proportion of surgical management of IMC compared to HGD and T1 cancer. Secondary outcomes were the adjusted odds ratios (OR) for surgical management, proportions of synchronous advanced neoplasia, and adjusted hazard ratios (HR) for metachronous advanced neoplasia.

**Results:**

We identified 455 patients with follow-up endoscopy and pathology (mean age 67.1y, 42.2% female, median follow-up 3.4y): 269 with HGD, 60 with IMC, 126 with T1 CRC. Compared to patients with HGD, patients with IMC were significantly more likely to receive a CT of the abdomen despite a complete endoscopic resection (52.6% vs 13.2%, p<0.001). Surgery rates were 15.2% for HGD, 36.7% for IMC and 84.1% for T1 CRC (p<0.001). When evaluating reasons for surgery, patients with IMC were more likely to undergo unnecessary surgery after a complete endoscopic resection compared to patients with HGD (22.7% of surgical resection reasons in IMC group vs 0% in HGD group, p<0.001). After adjusting for select factors potentially affecting the decision for surgical management and that differed between groups (age, family history of CRC, lesion location, size and morphology, multiplicity of synchronous lesions, colonoscopy indication/context), the adjusted odds of surgery remained significantly higher in patients with IMC compared to HGD (OR 2.62 [1.18-5.85]). Proportions of synchronous advanced neoplasia were 24.2% for HGD, 26.7% for IMC and 25.4% for T1 CRC (p=0.908). The proportion of metachronous advanced neoplasia was 26.8% in the HGD group, 18.3% in the IMC group and 20.6% in the T1 CRC group (p=0.227). Compared to HGD, patients with IMC and T1 CRC had similar adjusted metachronous advanced neoplasia risks (respectively, HR 0.82 [0.43-1.59] and HR 1.16 [0.66-2.05]). No lymph nodes were positive (0/363) and no metastasis occurred among patients with IMC.

**Conclusions:**

Patients diagnosed with colorectal IMC are more likely to receive imaging and surgery than when HGD is diagnosed, although they are not at increased risk of synchronous advanced neoplasia, metachronous advanced neoplasia, lymph node involvement or metastasis. Labeling colorectal lesions as IMC is associated with unnecessary surgical resections.

**Funding Agencies:**

TRIANGLE

